# Supplementary material for: Retinal Alterations as Potential Biomarkers of Structural Brain Changes in Alzheimer’s Disease Spectrum Patients
Source: Brain Sci. 2023 Mar 8;13(3):460. doi: 10.3390/brainsci13030460 (PMC10046312; doi:10.3390/brainsci13030460)
Supplement: Supplementary file 1 [file brainsci-13-00460-s001.zip › brainsci-2169313-supplementary.pdf]

Table S1. Comparison of retinal measures between cognitively normal group and cognitive impairment group.

Items	CN (21)	CI (27)	t	*p*-value
Vessel Density				
Center Ring	5.44 ± 2.63	3.8 ± 3.08	0.495	0.486
Inner Ring	13.87 ± 3.07	13.11 ± 3.69	0.705	0.407
Outer Ring	15.4 ± 2.47	14.42 ± 3.13	2.865	0.099
Full Ring	14.75 ± 2.51	13.65 ± 3.47	2.146	0.152
TI	13.52 ± 3.63	13.61 ± 3.01	0.635	0.431
SI	16.45 ± 12.82	12.74 ± 4.08	1.172	0.286
NI	14.16 ± 2.88	12.86 ± 3.34	0.449	0.507
II	13.91 ± 3.36	13.91 ± 2.94	0.091	0.765
TO	13.59 ± 3.3	13.81 ± 2.93	0.485	0.491
SO	15.03 ± 2.8	14.08 ± 3.58	3.005	0.092
NO	17.43 ± 2.75	16.39 ± 2.55	1.108	0.299
IO	15.48 ± 2.72	14.69 ± 2.76	4.144	0.049*
FAZ Area	0.23 ± 0.09	0.28 ± 0.16	0.026	0.874
RNFL Thickness				
Full	95 ± 8.23	92.5 ± 10.25	0.026	0.874
T	70.29 ± 13.72	70.13 ± 13.01	0.297	0.589
S	117.37 ± 15.22	103.3 ± 30.68	0.052	0.821
N	82.97 ± 43.85	65.93 ± 12.08	0.289	0.594
I	122.29 ± 14.49	118.2 ± 18.29	0.085	0.772
ILM-RPE Thickness				
TI	320.15 ± 14.46	317.5 ± 14.7	0.017	0.898
SI	280.15 ± 12.47	276.84 ± 9.54	0.030	0.864
NI	314.45 ± 14.58	311.94 ± 15.52	0.038	0.846
II	266.2 ± 12.35	266.35 ± 12.81	0.101	0.752
TO	307.2 ± 14.95	305.68 ± 14.44	0.187	0.668
SO	263.65 ± 14.41	264.26 ± 9.8	0.273	0.605
NO	320.4 ± 16.12	318.76 ± 16.06	0.017	0.897
IO	294.4 ± 12.87	294.94 ± 11.84	0.006	0.939

Data are expressed as the mean ± standard deviation (SD). All data were analyzed by covariance (ANCOVA), accounting for sex, age, years of education. CN: cognitively normal; CI: cognitive impairment; TI, temporal inner; TO, temporal outer; NI, nasal inner; NO, nasal outer; SI, superior inner; SO, superior outer, II, inferior inner, IO, inferior outer; FAZ, foveal avascular zone; RNFL, retinal nerve fiber layer; ILM-RPE, inner limiting membrane - retinal pigment epithelium. * < 0.05.

Table S2. Comparison of white matter integrity between cognitively normal group and cognitive impairment group.

Items	CN (21)	CI (27)	F	*p*-value
**FA**				
ATR	0.42 ± 0.03	0.43 ± 0.026	3.27	0.148
CST	0.6 ± 0.04	0.6 ± 0.028	0.35	0.704
CgC	0.54 ± 0.04	0.53 ± 0.043	0.11	0.896
CgH	0.48 ± 0.04	0.46 ± 0.039	1.77	0.183
Fma	0.57 ± 0.03	0.56 ± 0.033	0.37	0.692
IFO	0.49 ± 0.02	0.47 ± 0.035	0.03	0.970
ILF	0.47 ± 0.02	0.46 ± 0.03	0.22	0.800
SLF	0.46 ± 0.04	0.47 ± 0.026	1.83	0.174
UF	0.48 ± 0.03	0.46 ± 0.032	0.85	0.434
tSLF	0.5 ± 0.03	0.49 ± 0.06	2.24	0.119
**MD**				
ATR	0.0008 ± 0.000074	0.00081 ± 0.000079	0.65	0.426
CST	0.00072 ± 0.000031	0.00072 ± 0.000026	0.03	0.875
CgC	0.00074 ± 0.000032	0.00076 ± 0.000044	0.35	0.559
CgH	0.00073 ± 0.000046	0.00077 ± 0.000051	4.25	0.046*
Fma	0.00078 ± 0.000029	0.00079 ± 0.000046	0.06	0.807
IFO	0.00078 ± 0.000031	0.0008 ± 0.000048	0.04	0.834
ILF	0.00079 ± 0.000028	0.00081 ± 0.000053	0.28	0.597
SLF	0.00075 ± 0.000047	0.00075 ± 0.000033	0.43	0.515
UF	0.00076 ± 0.00003	0.00078 ± 0.00005	0.05	0.833
tSLF	0.00078 ± 0.000029	0.00079 ± 0.000049	0.35	0.555
**AxD**				
ATR	0.00118 ± 0.000077	0.00121 ± 0.000089	0	1
CST	0.00128 ± 0.000041	0.00128 ± 0.000026	0	1
CgC	0.00123 ± 0.000054	0.00125 ± 0.000055	0.295	0.59
CgH	0.00115 ± 0.000062	0.00119 ± 0.000056	5.043	0.03*
Fma	0.00136 ± 0.000039	0.00138 ± 0.000041	0.953	0.335
IFO	0.00124 ± 0.000033	0.00125 ± 0.000042	0.026	0.873
ILF	0.00123 ± 0.000042	0.00126 ± 0.000056	1.19	0.282
SLF	0.00114 ± 0.000043	0.00116 ± 0.00004	0.691	0.411
UF	0.00121 ± 0.000039	0.00121 ± 0.000054	0.01	0.922
tSLF	0.00125 ± 0.000054	0.00126 ± 0.000086	0.178	0.676
**RD**				
ATR	0.00061 ± 0.000076	0.00061 ± 0.000076	1.43	0.239
CST	0.00044 ± 0.000041	0.00044 ± 0.000035	0.03	0.872
CgC	0.0005 ± 0.00004	0.00052 ± 0.000054	0.16	0.691
CgH	0.00052 ± 0.000047	0.00057 ± 0.000057	2.55	0.118
Fma	0.00048 ± 0.000037	0.0005 ± 0.000054	0.02	0.885
IFO	0.00055 ± 0.000034	0.00057 ± 0.000056	0.04	0.838
ILF	0.00057 ± 0.000028	0.00059 ± 0.000056	0.02	0.895

SLF	0.00056 ± 0.000057	0.00055 ± 0.000036	1.53	0.224
UF	0.00053 ± 0.000037	0.00056 ± 0.000054	0.11	0.739
tSLF	0.00054 ± 0.000029	0.00056 ± 0.000059	0.22	0.638

Data are expressed as the mean ± standard deviation (SD). All data were analyzed by covariance (ANCOVA), accounting for sex, age, years of education. Abbreviations: CN: cognitive normal; CI: cognitive impairment; CgH: Cingulum hippocampus; UF: Uncinate fasciculus; FA: fractional anisotropy; MD: mean diffusivity; AxD: Axial Diffusivity; RD: Radial Diffusivity. ATR: anterior thalamic radiation; CgC: cingulum in the cingulated cortex area; CgH: cingulum in the hippocampal area; CST: cortitospinal tract; FMa: forceps major; IFO: inferior fronto-occipital fasciculus; ILF: inferior longitudinal fasciculus; SLF: superior longitudinal fasciculus; tSLF: the temporal projection of the SLF; UF: uncinate fasciculus. * < 0.05.

Table S3. Relationship Between hippocampal subfield volumes and retinal parameters in CI group.

Partial Correlation

Control	Items	Subiculum	Presubiculum	Molecular_layer_HP	GC-ML-DG	Whole hippocampus
Age, Gender, Years of education, eTIV	**Vessel density**					
	Center Ring	0.142	0.417	0.051	0.060	0.157
	Inner Ring	0.017	0.250	−0.057	0.019	0.024
	Outer Ring	0.293	0.435*	0.257	0.310	0.305
	Full Area	0.220	0.398	0.170	0.231	0.231
	FAZ Area	0.041	0.252	−0.009	−0.001	−0.008
	VD_TI	0.245	0.400	0.108	0.168	0.175
	VD_SI	0.150	0.438	0.062	0.044	0.130
	VD_NI	−0.033	0.176	−0.152	−0.129	−0.084
	VD_II	0.198	0.356	0.189	0.189	0.197
	VD_TO	0.196	0.300	0.155	0.174	0.180
	VD_SO	0.523*	0.593**	0.463*	0.452*	0.487*
	VD_NO	0.440	0.559*	0.339	0.301	0.348
	VD_IO	0.142	0.417	0.051	0.060	0.157
	**Thickness**					
	RNFL	0.264	0.160	0.317	0.271	0.284
	RNFL_T	0.011	−0.113	−0.039	−0.099	−0.056
	RNFL_S	0.235	0.162	0.277	0.267	0.236
	RNFL_N	0.276	0.176	0.357	0.339	0.329
	RNFL_I	0.215	0.253	0.291	0.274	0.296
	ILM-RFE_TI	0.303	0.336	0.299	0.248	0.355
	ILM-RFE_SI	0.309	0.287	0.293	0.224	0.350
	ILM-RFE_NI	0.307	0.278	0.312	0.234	0.363
	ILM-RFE_II	0.388	0.381	0.342	0.273	0.375
	ILM-RFE_TO	0.222	0.218	0.223	0.192	0.209
	ILM-RFE_SO	0.198	0.157	0.205	0.134	0.187
	ILM-RFE_NO	0.284	0.258	0.297	0.207	0.274
	ILM-RFE_IO	0.009	0.028	0.042	−0.008	0.039

Partial correlation was conducted controlling for age, gender, years of education, and eTIV. FAZ: foveal avascular zone; TI: temporal inner; TO: temporal outer; NI: nasal inner; NO: nasal outer; SI: superior inner; SO: superior outer: II: inferior inner, IO: inferior outer; VD: vessel density; RNFL: retinal nerve fiber layer; T: temporal, S: superior; N: nasal; I: inferior; ILM-RPE: inner limiting membrane - retinal pigment epithelium; GC-ML-DG: Molecular and Granule Cell Layers of the Dentate. * < 0.05.

Table S4. Relationship Between hippocampal subfield volumes and retinal parameters in CN group.

Partial Correlation

Control	Items	Subiculum	Presubiculum	Molecular_layer_HP	GC-ML-DG	Whole hippocampus
Age, Gender, Years of education, eTIV	**Vessel density**					
	Center Ring	0.200	0.548*	−0.041	0.105	−0.041
	Inner Ring	0.264	0.445	0.073	0.237	0.018
	Outer Ring	0.333	0.239	0.177	0.278	0.135
	Full Area	0.323	0.315	0.150	0.273	0.104
	FAZ Area	0.187	0.339	0.029	0.246	−0.017
	VD_TI	0.186	0.392	−0.066	0.081	−0.088
	VD_SI	0.305	0.502*	0.094	0.175	0.010
	VD_NI	0.328	0.442	0.233	0.377	0.175
	VD_II	0.216	0.250	0.115	0.323	0.106
	VD_TO	0.254	0.139	0.053	0.171	0.060
	VD_SO	0.261	0.166	0.106	0.053	0.039
	VD_NO	0.367	0.197	0.310	0.354	0.236
	VD_IO	0.200	0.548*	−0.041	0.105	−0.041
	**Thickness**					
	RNFL	−0.105	−0.210	0.030	−0.073	−0.028
	RNFL_T	−0.037	0.334	−0.046	−0.158	−0.163
	RNFL_S	−0.192	−0.202	−0.037	−0.105	−0.018
	RNFL_N	0.021	−0.334	0.235	0.200	0.198
	RNFL_I	−0.086	−0.307	−0.097	−0.139	−0.081
	ILM-RFE_TI	−0.084	0.196	0.081	0.015	−0.029
	ILM-RFE_SI	−0.098	0.148	0.014	−0.053	−0.078
	ILM-RFE_NI	−0.012	0.297	0.061	−0.088	−0.046
	ILM-RFE_II	0.013	0.133	0.198	0.087	0.075
	ILM-RFE_TO	−0.043	−0.072	0.093	0.160	0.027
	ILM-RFE_SO	−0.064	−0.052	0.097	0.097	0.017
	ILM-RFE_NO	−0.127	−0.150	0.147	0.017	0.021
	ILM-RFE_IO	−0.112	−0.157	0.231	0.155	0.136

Partial correlation was conducted controlling for age, gender, years of education, and eTIV. FAZ: foveal avascular zone; TI: temporal inner; TO: temporal outer; NI: nasal inner; NO: nasal outer; SI: superior inner; SO: superior outer: II: inferior inner, IO: inferior outer; VD: vessel density; RNFL: retinal nerve fiber layer; T: temporal, S: superior; N: nasal; I: inferior; ILM-RPE: inner limiting membrane - retinal pigment epithelium; GC-ML-DG: Molecular and Granule Cell Layers of the Dentate. * < 0.05.

Table S5. Relationship Between Diffusion Metrics and retinal parameters in CN group.

		Partial Correlation	
Control	Items	CgH (MD)	CgH (AxD)
Age, Gender, Years of education	**Vessel density**		
	Center Ring	−0.397	−0.194
	inner Ring	−0.072	−0.216
	Outer Ring	−0.235	−0.323
	Full Area	−0.156	−0.366
	FAZ Area	0.175	−0.013
	VD_TI	−0.268	−0.354
	VD_SI	−0.094	−0.217
	VD_NI	0.207	−0.135
	VD_II	−0.098	−0.100
	VD_TO	−0.174	−0.320
	VD_SO	−0.283	−0.210
	VD_NO	−0.272	−0.102
	VD_IO	−0.397	−0.194
	**Thickness**		
	RNFL	−0.158	0.108
	RNFL_T	0.115	0.205
	RNFL_S	−0.091	−0.033
	RNFL_N	−0.463	−0.138
	RNFL_I	−0.076	0.230
	ILM-RFE_TI	−0.331	0.015
	ILM-RFE_SI	−0.128	0.139
	ILM-RFE_NI	−0.287	−0.041
	ILM-RFE_II	0.121	0.147
	ILM-RFE_TO	−0.361	−0.147
	ILM-RFE_SO	−0.149	−0.004
	ILM-RFE_NO	−0.321	−0.068
	ILM-RFE_IO	−0.087	0.028

Partial correlation was conducted controlling for age, gender, years of education. FAZ: foveal avascular zone; TI: temporal inner; TO: temporal outer; NI: nasal inner; NO: nasal outer; SI: superior inner; SO: superior outer: II: inferior inner, IO: inferior outer; VD: vessel density; RNFL: retinal nerve fiber layer; T: temporal, S: superior; N: nasal; I: inferior; ILM-RPE: inner limiting membrane - retinal pigment epithelium; All data were analyzed via two-sample t-test. CN: cognitively normal; CI: cognitive impairment; CgH: Cingulum hippocampus; UF: Uncinate fasciculus; FA: fractional anisotropy; MD: mean diffusivity; AxD: Axial Diffusivity; RD: Radial Diffusivity. * $<$0.05.

Table S6. Relationship Between Diffusion Metrics and retinal parameters in CN group.

		Partial Correlation	
Control	Items	CgH (MD)	CgH (AxD)
Age, Gender, Years of education	**Vessel density**		
	Center Ring	−0.263	−0.334
	inner Ring	−0.387	−0.304
	Outer Ring	−0.205	−0.179
	Full Area	−0.267	−0.227
	FAZ Area	−0.397	−0.337
	VD_TI	0.454	0.495*
	VD_SI	−0.309	−0.225
	VD_NI	−0.383	−0.284
	VD_II	−0.350	−0.362
	VD_TO	−0.193	−0.223
	VD_SO	0.013	0.032
	VD_NO	−0.127	0.004
	VD_IO	−0.263	−0.334
	**Thickness**		
	RNFL	0.043	0.130
	RNFL_T	0.158	0.265
	RNFL_S	−0.290	−0.208
	RNFL_N	−0.007	0.057
	RNFL_I	−0.035	0.016
	ILM-RFE_TI	−0.043	0.141
	ILM-RFE_SI	0.058	0.126
	ILM-RFE_NI	0.015	0.143
	ILM-RFE_II	0.155	0.088
	ILM-RFE_TO	0.029	0.171
	ILM-RFE_SO	0.185	0.168
	ILM-RFE_NO	−0.015	0.114
	ILM-RFE_IO	0.186	0.238

Partial correlation was conducted controlling for age, gender, years of education. FAZ: foveal avascular zone; TI: temporal inner; TO: temporal outer; NI: nasal inner; NO: nasal outer; SI: superior inner; SO: superior outer: II: inferior inner, IO: inferior outer; VD: vessel density; RNFL: retinal nerve fiber layer; T: temporal, S: superior; N: nasal; I: inferior; ILM-RPE: inner limiting membrane - retinal pigment epithelium; All data were analyzed via two-sample t-test. CN: cognitively normal; CI: cognitive impairment; CgH: Cingulum hippocampus; UF: Uncinate fasciculus; FA: fractional anisotropy; MD: mean diffusivity; AxD: Axial Diffusivity; RD: Radial Diffusivity. * ＜0.05.

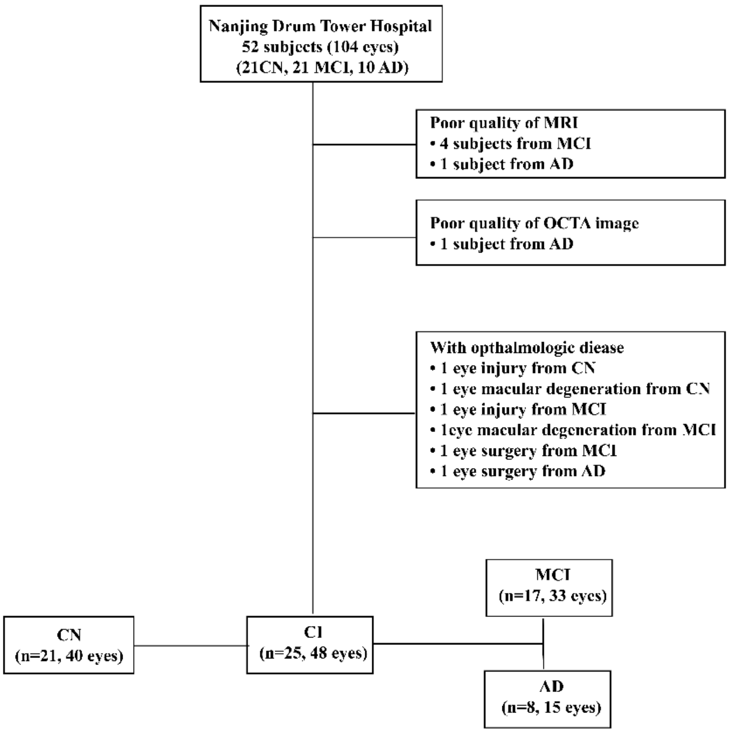

Figure S1. Flow Diagram of the Inclusion Process. CN, cognitively normal; MCI; mild cognitive impairment. AD, Alzheimer's Disease; CI; cognitive impairment. MRI, magnetic resonance imaging.

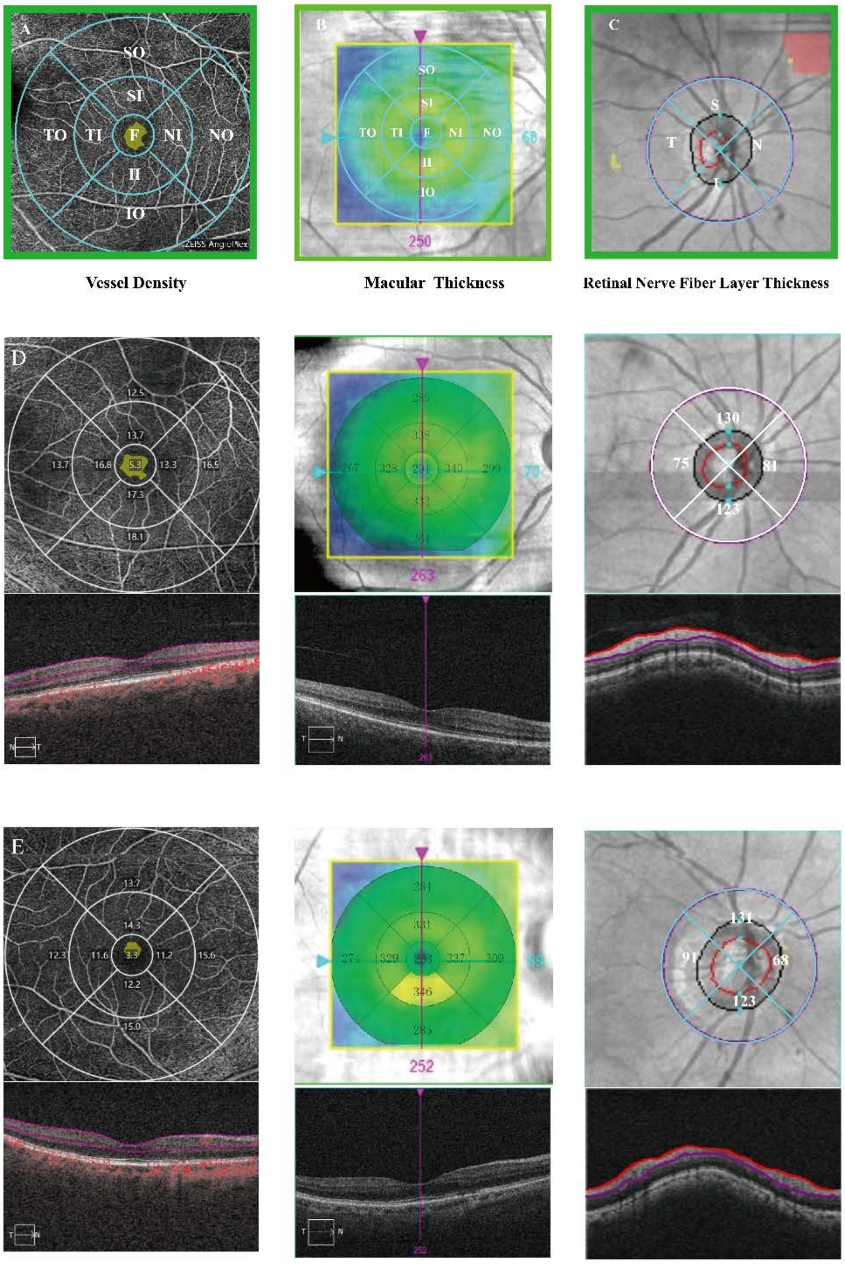

Figure S2. OCTA images. (A, B) In the macula, vessel density and retinal macular thickness are measured in the fovea (F) (Ø 1 mm), inner ring (Ø 3 mm), and outer ring (Ø 6 mm) following the Early Treatment Diabetic Retinopathy Study (ETDRS) grid. The inner ring and outer ring are subdivided into four sectors (superior, nasal, inferior, and temporal). (C) Around the optic nerve head (peripapillary): retinal nerve fiber layer (RNFL) thickness was measured in the following sectors: temporal (T), superior (S), nasal (N), inferior (I), and mean RNFL thickness (RNFL). (D) Retinal scan images and B-scans from healthy eye. (E) Retinal scan images and B-scans from patient eye.
